# Spatial mapping of chiral-induced spin selectivity in chiral perovskite via spin-Schottky junction

**DOI:** 10.1093/nsr/nwaf295

**Published:** 2025-07-21

**Authors:** Minghui Li, Zhongwei Chen, Xiting Lang, Junchuan Zhang, Yongjie Jiang, Hao Tian, Fang Ye, Xirui Liu, Yangyang Gou, Herui Xi, Wei Guo, Jichun Ye, Matthew C Beard, Haipeng Lu, Chuanxiao Xiao

**Affiliations:** Ningbo Institute of Materials Technology and Engineering, Chinese Academy of Sciences, Ningbo 315201, China; School of Materials Science and Chemical Engineering, Ningbo University, Ningbo 315211, China; Department of Chemistry, The Hong Kong University of Science and Technology, Hong Kong 999077, China; Ningbo Institute of Materials Technology and Engineering, Chinese Academy of Sciences, Ningbo 315201, China; Ningbo Institute of Materials Technology and Engineering, Chinese Academy of Sciences, Ningbo 315201, China; Ningbo Institute of Materials Technology and Engineering, Chinese Academy of Sciences, Ningbo 315201, China; Ningbo Institute of Materials Technology and Engineering, Chinese Academy of Sciences, Ningbo 315201, China; Ningbo Institute of Materials Technology and Engineering, Chinese Academy of Sciences, Ningbo 315201, China; School of Materials Science and Chemical Engineering, Ningbo University, Ningbo 315211, China; Ningbo Institute of Materials Technology and Engineering, Chinese Academy of Sciences, Ningbo 315201, China; School of Materials Science and Chemical Engineering, Ningbo University, Ningbo 315211, China; Ningbo Institute of Materials Technology and Engineering, Chinese Academy of Sciences, Ningbo 315201, China; Ningbo Institute of Materials Technology and Engineering, Chinese Academy of Sciences, Ningbo 315201, China; Ningbo Institute of Materials Technology and Engineering, Chinese Academy of Sciences, Ningbo 315201, China; Ningbo Institute of Materials Technology and Engineering, Chinese Academy of Sciences, Ningbo 315201, China; Chemistry & Nanoscience Center, National Renewable Energy Laboratory, Golden, CO 80401, USA; Department of Chemistry, The Hong Kong University of Science and Technology, Hong Kong 999077, China; Ningbo Institute of Materials Technology and Engineering, Chinese Academy of Sciences, Ningbo 315201, China; Ningbo New Materials Testing and Evaluation Center CO., Ltd, Ningbo 315201, China

**Keywords:** chiral-induced spin selectivity, Kelvin probe force microscopy, chiral halide perovskite, spin-Schottky junction, inhomogeneity

## Abstract

Chiral halide perovskite (c-HP) semiconductors exhibit on average a large chiral-induced spin selectivity (CISS) effect. Nevertheless, the microscopic details of CISS and its integration in opto-spintronic constructs remain nascent. Reliable reporting of CISS performance characteristics represents a significant challenge in providing the necessary design rules. We show a Kelvin probe force microscopy (KPFM) method that can quantitatively evaluate and spatially map the chirality-dependent surface contact potential difference resulting from the formation of a spin-Schottky junction. We revealed inhomogeneity in the CISS response, where low-CISS regions in the c-HP films reduce the overall macroscopic average, likely serving as a key factor in optimizing macroscopic performance. We also observed that although c-HP films made from higher precursor concentrations lead to thicker films and higher carrier concentrations with subsequent larger barrier heights in the Schottky junction, stronger spin relaxation due to non-ideal film quality reduces spin polarization.

## Teaser

This study uses advanced microscopy to map how chiral perovskites affect electron spin behavior, providing new insights into their microscopic properties through spin-Schottky junctions.

## INTRODUCTION

Harnessing the spin-degrees of freedom of charge carriers in semiconductor structures offers significant potential for a wide range of potential applications, e.g. enhancing the efficiency of data storage and transfer [1,2]. This approach is particularly promising in the fields of quantum computing and neuromorphic computing [3,4]. Key aspects include control over spin injection at dissimilar interfaces, spin accumulation, and spin-polarized current densities. A novel approach to achieve this control at room temperature is through the chiral-induced spin selectivity (CISS) effect [5,6]. CISS describes a process whereby when charge current transports through a chiral medium, the spin orientation of the charge carriers becomes polarized with either spin-up or -down configuration depending on the chirality and current direction. Thus, CISS can allow for modulation of spin populations without the need for magnetic components. The CISS effect has been demonstrated in various self-assembled monolayers exhibiting chirality, including helical deoxyribonucleic acid (DNA), oligopeptides and helicenes [7–12]. Notably, chiral halide-perovskite semiconductors (c-HPs), with chiral organo-ammonium cations oriented in an inorganic metal halide sublattice, have recently been shown to exhibit CISS in thin films [13–16]. Moreover, studies investigating the influence of external factors and compositional variations on the chiral quality of c-HPs are also on the rise [17,18]. The advent of c-HPs marks a new era, as it leverages thin film semiconductors rather than monolayers of chiral molecules at interfaces, allowing for the realization of CISS in semiconductor structures. Thus, c-HPs can enable control over charge, spin and light at room temperature without the need for magnetic components [19–23]. Since their discovery, c-HPs have been incorporated into a wide range of potential applications. For instance, Chen *et al.* fabricated a direct circularly polarized (CP) light photodetector using c-HP as both the light absorber and spin discrimator (*R*- and *S*-α-phenylethylamine)PbI_3_ and demonstrated impressive performance in CP light detection with high sensitivity, low noise and rapid response [24]. The incorporation of chiral organo-ammonium cations, such as *R*/*S*-α-methylbenzylammonium (*R*/*S*-MBA), in highly textured, crystalline c-HP thin films has enabled spin accumulation in an adjacent non-chiral semiconductor layer [25]. This was demonstrated through the development of spin light-emitting diodes (spin-LEDs) [26,27] and spin-photovoltaic structures [28]. Despite these promising developments, the field of c-HPs is still in its infancy and further mechanistic understanding is needed for them to reach their full potential.

To this end, it is critical to understand the chirality transfer mechanism, where the chirality inherent in the organic component is imprinted onto the inorganic component, and the impact of chiral transfer on CISS, especially in macroscopic semiconductor interfaces and heterostructures. However, there are barriers to uncovering microscopic design rules that govern CISS in c-HPs in order to fully take advantage of the effect. Characterization tools suitable for CISS evaluation remain limited. One pivotal characterization tool is magnetic conductive probe atomic force microscopy (mCP-AFM), which utilizes a small probe (usually 5–30 nm in radius) that operates in contact mode to measure magneto current–voltage (*I–V*) characteristics that demonstrate CISS [26,29–32]. The mCP-AFM applies continuously varying voltages to the sample and records current–voltage (*I–V*) curves to evaluate the CISS performance characteristics based on differences in the *I–V* characteristics for a particular magnetic polarization and chirality [25,33,34]. Another scanning probe microscopy (SPM) technique, Kelvin probe force microscopy (KPFM), operates in tapping mode and detects the contact potential difference (CPD) by continuously nullifying the electrostatic force between the probe and the sample surface. Ghosh *et al.* used KPFM to measure the changes in CPD to reflect the spin dependence of charge penetration from the ferromagnet (FM) into the adsorbed chiral molecular self-assembled layers [35]. Other related studies have also explored the potential of KPFM for CISS research, both in theoretical modeling and experimental applications [36–39]. Although these techniques are powerful, with mCP-AFM widely adopted in the CISS research community [40–46], the measurements have not leveraged the high spatial resolution mapping capability of SPM, leaving a gap in our understanding of CISS and the potential for further improvements. Moreover, there appear to be quantitative discrepancies between the high spin polarization measured in mCP-AFM measurements and the low overall spin polarization realized, to date, in macroscopic spin valves. These features make it unclear how to further increase the spin polarization.

In this study, we applied an advanced KPFM mapping technique [47,48] to characterize CISS in c-HP thin films by scanning the same area under varying magnetization conditions. Figure S1 illustrates the KPFM testing procedure. The precise measurement of CPD in our home-built KPFM allows for the quantitative evaluation of CISS by changing the magnetization conditions: north pole, south pole and without additional magnetization field. We propose that a spin-Schottky junction forms between the FM metal and the c-HP layer, where the Fermi level depends on chirality and magnetic polarization and is responsible for the differences in CPD. Thus, the height of the Schottky barrier contains information about CISS. Our microscopic method provides nanometer-scale mapping of the CISS uniformity. By comparing the relative CPD changes for c-HP with different precursor concentrations, we observed a decreased selectivity of spin-polarized electrons in thicker films made from denser precursors. The method of evaluating CISS using KPFM facilitates a comprehensive analysis of c-HP thin films, and potentially opens new opportunities for materials discovery and incorporation.

## RESULTS AND DISCUSSION

We designed the KPFM measurements to deconvolute the room-temperature CISS effect from Fermi level changes on the microscopic level. In KPFM, the compensated voltage is applied to the probe, with the CPD representing the difference between the probe work function and the sample's surface work function (specifically, $q \cdot CPD$ = $\emptyset $_Tip_ − $\emptyset $_Sample_, where $\emptyset $ is the work function and *q* is the charge of an electron). Changes in CPD reflect variations in the sample's work function and electron Fermi level, assuming no surface states significantly affect the signal. Notably, a higher surface potential difference of the tested sample corresponds to a lower work function [49,50]. We first performed KPFM tests on the chiral (MBA)_2_PbI_4_ and Au both on silicon substrates, respectively.


(1) \begin{eqnarray*}
q \cdot CP{D}_{{\mathrm{Au}}} = {\emptyset }_{{\mathrm{Tip}}} - {\emptyset }_{{\mathrm{Au}}}
\end{eqnarray*}

(2) \begin{eqnarray*}
q \cdot CP{D}_{{\mathrm{c}} - {\mathrm{HP}}} = {\emptyset }_{{\mathrm{Tip}}} - {\emptyset }_{{\mathrm{c}} - {\mathrm{HP}}}
\end{eqnarray*}

Our results show a lower CPD on Au, indicating a lower Fermi level in Au compared to the c-HP semiconductor. Figures S2 and S3 also show that the c-HP materials are n-type semiconductors. The significant disparity in the respective work functions results in the formation of a Schottky junction at the (MBA)_2_PbI_4_–Au interface. *I–V* tests were performed on c-HP films spin-coated on FM substrates. The test results further confirm the presence of such a Schottky barrier (Fig. S4). The barrier height ($q{V}_{{\mathrm{bi}}}$) on the chiral n-type semiconductor side can be estimated as:


(3) \begin{eqnarray*}
q{V}_{{\mathrm{bi}}} = {\emptyset }_{{\mathrm{Au}}} - {\emptyset }_{{\mathrm{c}} - {\mathrm{HP}}} = {\emptyset }_{{\mathrm{Au}}} - {E}_0 + {E}_{\mathrm{F}},
\end{eqnarray*}

where ${\emptyset }_{{\mathrm{Au}}}$ and ${\emptyset }_{{\mathrm{c}} - {\mathrm{HP}}}$ are the work function of Au and c-HP, respectively, *E*_0_ is the vacuum energy level of the c-HP and *E*_F_ is the Fermi level.

**Figure 1.**
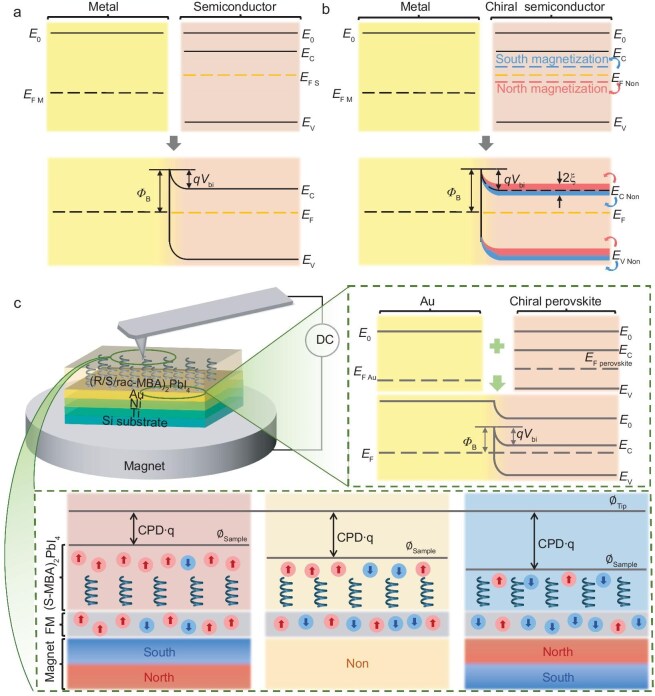
Schottky junctions, spin-Schottky junctions and experimental schematics. (a) Energy band diagram of a metal and a semiconductor in contact and forming a Schottky junction; (b) changes in spin-Schottky junctions affected by magnetization directions; (c) schematic of the KPFM experimental setup and principle of detecting CISS. The diagram illustrates the formation of spin-Schottky contact between the Au layer and the chiral perovskite film and changes in the work function under different magnetic polarizations. *E*_0_, *E_C_, E*_F_ and *E*_V_ refer to vacuum energy level, conduction band, Fermi level and valence band, respectively. ${\emptyset }_{\mathrm{B}}\ $is the barrier height on the metal side, $q{V}_{{\mathrm{bi}}}$ is the barrier height on the chiral n-type semiconductor side, ∅_sample_ is the work function of the sample, $\chi $ is the electron affinity and $\xi \ $is the difference in the spin-dependent Fermi level induced by the CISS effect. ‘North’ refers to the magnet's north pole, ‘South’ refers to the magnet's south pole, and ‘Non’ refers to the condition of no magnetic field.

The formation of the Schottky junction results in an internal electrical field that gives rise to charge polarization at the interface. However, when the semiconductor is chiral, electron spins should also be polarized during the Fermi-level equilibration due to the CISS effect that locks the spin orientation to the charge carrier motion, forming a spin-Schottky junction, that is the barrier height is different for the different spin states. The difference in the Schottky barrier height is a result of spin accumulation within the depletion region of the Schottky junction. Figure 1a shows the energy band diagram of a metal/semiconductor heterostructure and the formation of a Schottky junction, and Fig. 1b illustrates the formation and change of a spin-Schottky junction under different magnetic field conditions. It should be noted that the shifts in the conduction and valence bands in Fig. 1b result from aligning the Fermi levels under different magnetic environments. Due to the Fermi-level equilibration, spin-dependent work function and CISS, we expect spin accumulation within the depletion region of the Schottky junction. We envision that this spin-Schottky junction can be manifested under an external magnetic field, where spin selectivity through the c-HP will be observed.

To probe this, we prepare a series of spin-Schottky junction samples consisting of FM substrate and chiral/achiral HP thin films. The FM layer with the configuration of Ti 8 nm/Ni 80 nm/Au 8 nm was deposited on the silicon wafer by electron beam deposition, in which a layer of Au is necessary to protect the FM Ni. Perovskite semiconductors, (*R*-MBA)_2_PbI_4_, (*S*-MBA)_2_PbI_4_ or (*rac*-MBA)_2_PbI_4_, were spin-coated on the FM substrate. Figure 1c shows the experimental setup for KPFM testing under magnetization conditions and the formation of spin-Schottky junction between the Au layer and c-HP thin film. The completed sample was placed atop a strong magnet, and the magnetic polarization of the FM was altered by flipping the permanent magnet. The strength of the magnetic field was also measured (Fig. S5).

The work function, or measured CPD value, is affected by both the chirality and the magnetic polarization of the FM substrate [35]. The work function represents the minimum energy required to immediately remove an electron from the solid. The FM substrate offers spin-up and spin-down oriented electrons at the Fermi level, which will display different transport characteristics according to the CISS mechanism. Chiral perovskite semiconductors serve as selective transport media, allowing electrons with a specific spin orientation to pass more readily, while those with the opposite spin orientation encounter greater hindrance. When the number of spin-polarized electrons increases, the work function decreases and vice versa. The new barrier height on the chiral semiconductor side under different magnetization conditions can be estimated as:


(4) \begin{eqnarray*}
q{V}_{{\mathrm{bi\ North}}} = {\mathrm{\ }}{\emptyset }_{{\mathrm{Au}}} - {E}_0 + {E}_{{\mathrm{F\ North}}},
\end{eqnarray*}

(5) \begin{eqnarray*}
q{V}_{{\mathrm{bi\ South}}} = {\mathrm{\ }}{\emptyset }_{{\mathrm{Au}}} - {E}_0 + {E}_{{\mathrm{F\ South}}},
\end{eqnarray*}

where ${\emptyset }_{{\mathrm{Au}}}$ is the work function of Au, and ${E}_0$ is the vacuum energy level of chiral (MBA)_2_PbI_4_ perovskite. ${E}_{{\mathrm{F\ North}}}$ and ${E}_{{\mathrm{F\ South}}}$ represent the Fermi levels under north and south magnetization, respectively. The change in spin-polarized electrons and work function is illustrated in Fig. 1c. As shown in Fig. 1c, due to the influence of the CISS effect, the *E*_F_ of (*S*-MBA)_2_PbI_4_ under south-pole magnetization is higher than that under north-pole magnetization, indicating the presence of a spin-Schottky junction in the sample. We define the difference ξ as the Fermi level offset of the (MBA)₂PbI₄ material under different magnetization conditions:


(6) \begin{eqnarray*}
\xi = {E}_{\rm F\,{\mathrm{North}}} - {E}_{\rm F\,{\mathrm{South}}} = {\emptyset }_{c - {\mathrm{HP\,North}}} - {\emptyset }_{c - {\mathrm{HP\,South}}},
\end{eqnarray*}

where ${\emptyset }_{{\mathrm{c}} - {\mathrm{HP\ North}}}$ and ${\emptyset }_{{\mathrm{c}} - {\mathrm{HP\ South}}}$ are the work functions of c-HP under north and south magnetization conditions, respectively. Since KPFM can evaluate changes in the sample's work function through CPD, we can derive the following:


(7) \begin{eqnarray*}
\xi = q \cdot \left( {CP{D}_{{\mathrm{North}}} - CP{D}_{{\mathrm{South}}}} \right)\ = \ q \cdot \Delta {\mathrm{PP}},
\end{eqnarray*}

where *q* is the charge of an electron, and $CP{D}_{{\mathrm{North}}}$ and $CP{D}_{{\mathrm{South}}}$ are the CPDs measured under north- and south-oriented magnetization, respectively. ∆PP is the potential peak value difference. For the racemic HP, no change in CPD is observed, indicating the absence of CISS. Therefore, the CISS effect in c-HP thin films can be evaluated and quantified by the CPD difference observed due to the flipping of the underlying magnetization directions.

**Figure 2.**
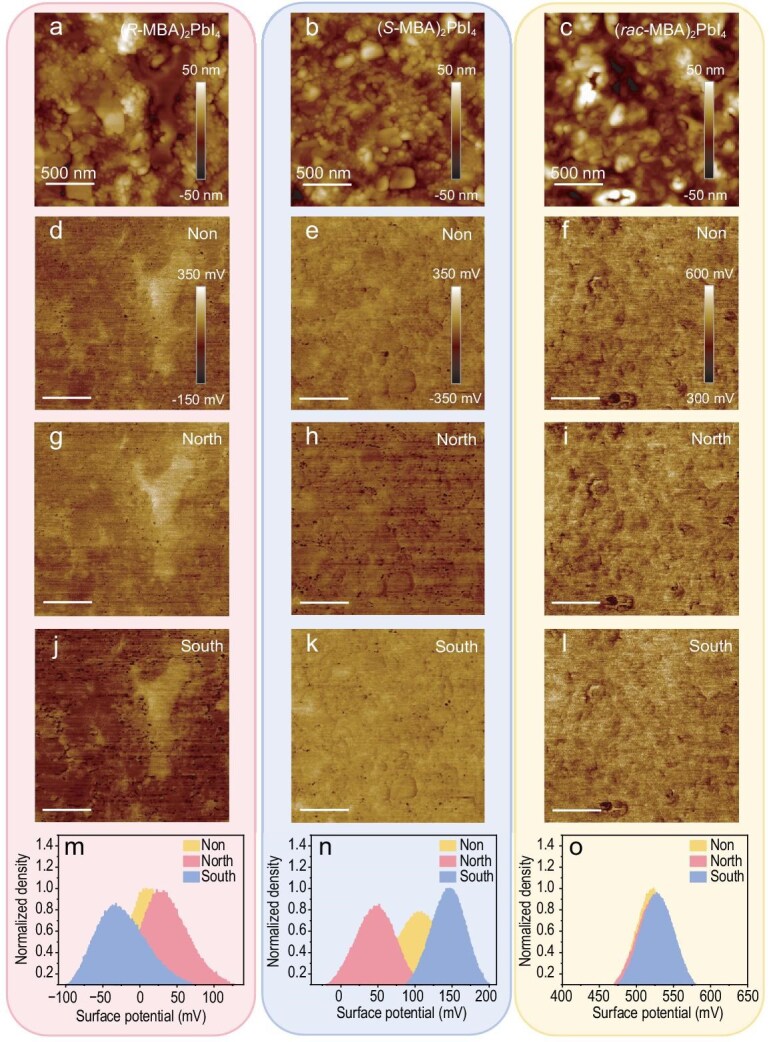
KPFM images and histograms of different chiral halide perovskites. (a–c) Topography images of (*R*-MBA)_2_Pbl_4_, (*S*-MBA)_2_Pbl_4_ and (*rac*-MBA)_2_Pbl_4_ samples, respectively. (d–f) Surface potential images under no additional magnetization field of (*R*-MBA)_2_Pbl_4_, (*S*-MBA)_2_Pbl_4_ and (*rac*-MBA)_2_Pbl_4_ samples, respectively, N-pole magnetic field (g–i) and S-pole magnetic field (j–l). (m–o) Normalized surface potential distribution histogram in different conditions. The scale bar is 500 nm.

Figure 2 shows the most representative results of c-HP films on Au/Ni substrates under different magnetic fields. The same location was analyzed under varying magnetic field conditions, with the location determined based on identical topography. Figure 2a–c shows AFM topography results obtained from randomly selected regions on thin film samples of (*R*-MBA)_2_PbI_4_, (*S*-MBA)_2_PbI_4_ and (*rac*-MBA)_2_PbI_4_ at a concentration of 0.1 mg/μL. The AFM images reveal the surface morphology of the chiral semiconductors at the nanometer scale, indicating that the c-HP uniformly covers the substrate. As shown in the scanning electron microscope (SEM) images in Figs S6–S8, it also demonstrates that the c-HP layer is effectively covering the substrate with no pinholes. Figures S9 and S10 further reveal the 2D perovskite phase structure and the chiral properties of the c-HP. However, the surface of the films exhibits inhomogeneity with varying domain sizes, likely attributed to different crystallization dynamics. In Fig. 2, the absolute CPD value of (*rac*-MBA)_2_PbI_4_ is greater than that of (*R*-MBA)_2_PbI_4_ and (*S*-MBA)_2_PbI_4_, due to the use of different probes during scanning. As shown in Equation (2), $\emptyset $_Tip_ affects the $CP{D}_{{\mathrm{c}} - {\mathrm{HP}}}$ results but does not impact our study of CISS. By measuring the CPD results at the same positions for the different samples under three magnetic field conditions, namely no additional magnetization field (Fig. 2d–f), north- (Fig. 2g–i) and south-pole magnetization (Fig. 2j–l), we can analyze the CISS effect in a fast and reliable manner. To facilitate the analysis, the CPD results under different magnetization conditions are placed on the same scale. Brighter KPFM images correspond to higher surface potentials and vice versa. A comparison between Fig. 2g and j reveals that the brightness of the (*R*-MBA)_2_PbI_4_ films is more pronounced under north-pole magnetization, indicating that the CPD in north-pole magnetization is higher than that in south-pole magnetization. In contrast, (*S*-MBA)_2_PbI_4_ film exhibits a higher CPD in south-pole magnetization. Conversely, the results for the (*rac*-MBA)_2_PbI_4_ show nearly identical CPD values across the different magnetization conditions (Fig. 2f, i and l). These results indicate that the chirality plays a critical role in selectively transporting spin-polarized electrons, with significant CPD offset observed under different magnetization conditions.

The normalized potential histogram and the potential peak value difference (∆PP) derived from the KPFM mapping can provide statistical insights for quantitative evaluation of CISS in chiral thin films. By subtracting the potential peak values under north-pole magnetization from those under south-pole magnetization in the histogram, ∆PP is obtained. Changes in ∆PP can quantify the overall offset of the potential peaks, and is a direct indication of the spin-dependent Fermi level change, denoted as $\xi $. Figure 2m–o shows the normalized potential histogram of *R*-, *S*- and *rac*-perovskites. ∆PP values of 60 mV for (*R*-MBA)_2_PbI_4_ and −95 mV for (*S*-MBA)_2_PbI_4_ indicate a more pronounced CISS effect in the latter. Meanwhile, the opposite sign of ∆PP indicates the spin selectivity is opposite for *R*- and S- c-HP. The normalized potential histogram of (*rac*-MBA)_2_PbI_4_ materials nearly overlaps under both north- and south-pole magnetizations, with no change in CPD observed in Fig. 2o, indicating the absence of spin selectivity. These findings demonstrate that ∆PP can identify the specific interaction between the FM substrate and the c-HP and simultaneously provide a quantitative assessment of CISS.

The distinct behaviors of ∆PP under varying magnetic fields originate from the interaction between the FM substrate and the chiral semiconductors. The surface potential peak shifts under different conditions can be attributed to the interplay between spin-polarized electrons in the FM substrate under magnetization conditions and the CISS effect of c-HP film. Upon magnetization of the FM substrate, there is a spin accumulation at the interface. Spin-polarized electrons that are more compatible with (*R*-MBA)_2_PbI_4_ can be efficiently transported under north-pole magnetization conditions. These spins of the carriers are selectively transported through chiral molecules, subsequently modifying the electronic states on the surface of c-HP film. This leads to an increase in the Fermi level and a corresponding increase in measured CPD. However, spin electrons under south-pole magnetization encounter increased resistance from chiral molecules during transport, hindering their transfer to the surface. This results in a lower Fermi level, corresponding to a decrease in CPD. Under no additional magnetization field, the magnetic polarization is random and the distribution of electrons with different spin directions in the FM substrate is the same. Therefore, the number of spin-polarized electrons selectively transported by chiral molecules lies between the two magnetization conditions, resulting in a CPD peak between north- and south-pole magnetization conditions. (*S*-MBA)_2_PbI_4_ materials exhibit the opposite trend to (*R*-MBA)_2_PbI_4_, owing to the opposite chiral conformation. This phenomenon is determined by the distinct chiral selectivity of the two c-HP thin films. The results for the (*rac*-MBA)_2_PbI_4_ thin film indicate that the transport of spin-polarized electrons is not affected, and there are no observable changes in Fermi level and work function. Consequently, ∆PP remains close to zero and CPD does not vary with the magnetic fields.

The presented KPFM method offers several advantages over the traditional mCP-AFM [25,26,30,31]. Unlike mCP-AFM, which operates in contact mode, KPFM employs tapping mode. This distinction is crucial when dealing with the soft perovskite semiconductors to prevent scratching and to minimize contact resistance. Additionally, mCP-AFM requires either the probe or the substrate to be magnetized, and the voltage must be ramped at each point. This makes the mapping process time-consuming and challenging, especially when rescanning the same location. Although mCP-AFM provides a more direct approach to understanding CISS, we believe that the KPFM method proposed here offers a faster and more reliable way to characterize CISS.

**Figure 3.**
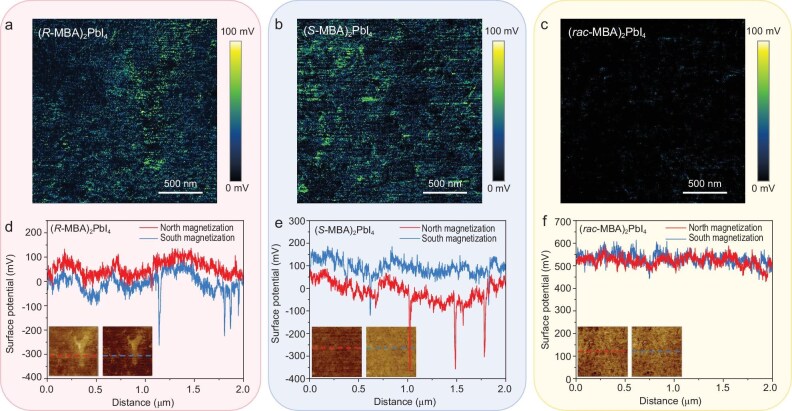
KPFM difference distribution images and profiles under different magnetic fields. (a–c) The CPD difference between the north-pole and south-pole magnetizations of (*R*-/*S*-/*rac*-MBA)_2_Pbl_4_; (d–f) line profiles of (*R*-/*S*-/*rac*-MBA)_2_Pbl_4_. The dashed lines in the inset indicate where the line profiles were taken.

To illustrate this, we took advantage of the mapping capability to visualize the spatial uniformity and homogeneity of the surface potential under varying conditions. Since the KPFM mappings under north- and south-pole magnetizations were conducted at the same location, we carefully aligned their topography images and subtracted the two potential images pixel by pixel [48,51] to acquire CPD difference distribution images for (*R*-MBA)_2_PbI_4_ and (*S*-MBA)_2_PbI_4_ thin films (Fig. 3a and b). The normalized potential histograms in Fig. 2m and n demonstrate statistical surface potential shifts for the two c-HPs under different magnetization conditions, with (*S*-MBA)_2_PbI_4_ exhibiting a wider variation. In Fig. 3a and b, we reveal that the spin electron transport on the plane of c-HP film is inhomogeneous, with the CPD difference ranging from 10 to 100 mV. These differences can be attributed to the varying contributions of microscale non-uniformity to the transport of spin-polarized carriers. Figure 3d and e depicts the potential line profiles at the same positions for (*R*-MBA)_2_PbI_4_ and (*S*-MBA)_2_PbI_4_ under different magnetization conditions, providing another perspective on the existence of spatial uniformity differences. These localized potential fluctuations arise from inhomogeneous transport. Figure 3c and f present the CPD potential difference results and line profiles for the (*rac*-MBA)_2_PbI_4_ sample, indicating that the sample does not exhibit the CISS effect. Figure S11 shows additional line profiles from the KPFM mappings. These results are consistent with the analysis in Fig. 3d–f, indicating the prevalence of CPD inhomogeneity.

Currently, effectively characterizing and utilizing CISS on the macroscopic level remains a significant challenge, even though c-HPs have been shown to exhibit a spin polarization of over 90% [25] in mCP-AFM. The nanometer-scale non-uniformity revealed in the KPFM (Fig. 3) offers important insights into this inconsistency. Analogous to the ‘Bucket effect’, the weakest point of CISS in the film limits its effect on the macroscopic level, i.e. the spin polarization is short circuited. For instance, while the CISS effect can be reflected through *I–V* characteristics, this commonly used testing method often yields much stronger results compared to macroscopic findings. In this case, the macroscopic *I–V* results indicate that the spin polarization is only 10%–20% (Fig. S4). This large inconsistency is because the measured *I–V* characteristics are affected by regions with weak spin selectivity, with the equivalent resistance being determined by these lower spin-polarized leakage points. Hence, the macroscopic results do not represent the average spin polarization but instead the minimum polarization. The KPFM method presented here offers significant advantages in characterizing CISS. For example, our work shows that sample uniformity across the entire c-HP film is essential to bridge the gap between excellent microscopic CISS and macroscopic performance characteristics. For the inhomogeneity of chiral polycrystalline perovskites, the mapping images capabilities of the KPFM enable a more comprehensive nanoscale evaluation of their CISS effect, while also enhancing our understanding of the underlying mechanisms.

**Figure 4.**
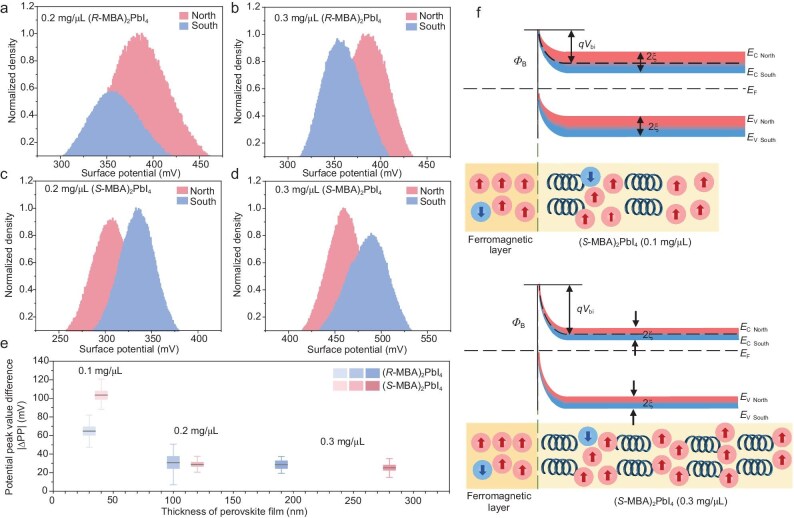
KPFM result histogram of CISS effect and spin-Schottky junction. Histogram of KPFM results of CISS effect on the same location of (a) (*R*-MBA)_2_Pbl_4_ (0.2 mg/μL), (b) (*R*-MBA)_2_Pbl_4_ (0.3 mg/μL), (c) (*S*-MBA)_2_Pbl_4_ (0.2 mg/μL) and (d) (*S*-MBA)_2_Pbl_4_ (0.3 mg/μL) samples. (e) The absolute potential difference of perovskite samples with various thicknesses. (f) The energy level changes for chiral perovskite with different thicknesses.

To further investigate CISS, we performed the KPFM experiments on samples with different thicknesses made from various concentrations (0.2–0.3 mg/μL). The KPFM results are similar to those obtained from 0.1 mg/μL samples (Figs S12 and S13), revealing non-uniform spin polarization. The normalized potential histogram reveals that for (*R*-MBA)_2_PbI_4_ prepared from higher concentrations, spin-polarized carriers remain more easily transported under north-pole magnetization conditions (Fig. 4a and b), while the transport behavior of (*S*-MBA)_2_PbI_4_ (Fig. 4c and d) remains opposite to that of (*R*-MBA)_2_PbI_4_. We analyzed the half-width at half maximum (HWHM) of the normalized potential peaks to gain further insights into the CPD distributions (Tables S1 and S2). We found that the HWHM of the normalized potential peaks measured in chiral molecule-selective magnetization conditions is mostly narrower than that in the non-selective magnetization conditions. We conducted SEM cross-sectional tests on different concentrations of (*R*-MBA)_2_PbI_4_ and (*S*-MBA)_2_PbI_4_ to determine the film thickness of the c-HP layer (Figs S14 and S15). In Fig. 4e, we show that as the concentration increases from 0.1 to ∼0.3 mg/μL, the thickness of the c-HP layer increases non-linearly, while the absolute ∆PP shows a noticeable decrease. Specifically, the absolute ∆PP for (*R*-MBA)_2_PbI_4_ decreases from an initial 60 mV to 30 mV, while the absolute ∆PP for (*S*-MBA)_2_PbI_4_ materials decreases more rapidly, from an initial 95 mV to 30 mV. The measurement uncertainty is represented by the error bars. However, it is evident that CISS in c-HP weakens with increasing precursor concentration. On one hand, we observed an increase in CPD from these thicker films deposited directly on a silicon substrate without the FM layer (Figs S16 and S17). The results indicate that the *E*_F_ shifts towards the conduction band and tends to be more n-type with denser precursor concentrations, resulting in a larger barrier height in the Schottky junction. With a certain number of spin-filtered electrons, the more n-type c-HP with higher donor concentrations can reduce the proportion of spin-polarized carriers, resulting in a relatively small CISS effect. On the other hand, while the denser precursor results in thicker films, they likely also modify the film growth process. The overall reduced CISS suggests that due to non-ideal film growth, spin relaxation may dominate in competition with the enhanced spin selectivity from the chiral barrier layers [25,28]. Additionally, spin dephasing at the spin-Schottky interface will also contribute to the final spin polarization. Therefore, the combination of spin selectivity, carrier concentration and spin relaxation can influence the exhibited CISS effect. Optimizing the film growth parameters and promoting interface engineering research will further extend the spin dephasing lifetime and enhance CISS performance.

Figure 4f illustrates a schematic of the spin-Schottky junction formed by the FM layer and c-HP with various concentrations. Traditionally, the difference between the work function of the FM layer and the electron affinity of the perovskite semiconductor determines $q{V}_{{\mathrm{bi}}}$ of the Schottky junction. However, in terms of c-HP, the chirality of molecules, the magnetic field directions and the strength of the CISS effect alter its Fermi level, leading to a change in *qV*_bi_. Schottky diodes have been widely used in numerous applications for either very low-power signal detection or high-power rectification, as well as building blocks for various electronic devices, such as photodiodes and transistors. In the spin-Schottky junction, the barrier height can be tuned and results in polarization of both carrier and carrier spin at the interface. We believe that the formation of spin-Schottky junctions paves the way for new opportunities in spintronic applications. For instance, a spin diode should also be possible between chiral n- and p-type semiconductors, which will enable both charge and spin polarization.

## CONCLUSION

In conclusion, we developed a KPFM method to quantitatively evaluate and map CISS on a microscopic scale within c-HP thin films. We proposed that a spin-Schottky junction forms between the FM layer and c-HP layer. The quantitative evaluation method using KPFM can probe the same position on the c-HP layer under different magnetization conditions, and the different CPD under different magnetization is attributed to CISS in the c-HP. The presence of spin selectivity leads to variations in Fermi level and resulting CPD under varying magnetization conditions. The KPFM yielded normalized CPD histograms and the CPD overall offset parameter ∆PP, which is directly related to the CISS strength for (*R*-MBA)_2_PbI_4_ and (*S*-MBA)_2_PbI_4_. The KPFM mapping can visualize the non-uniformity in ∆PP at the nanometer length scale. We conducted tests on three different precursor concentrations of (*R*-MBA)_2_PbI_4_ and (*S*-MBA)_2_PbI_4_, and found that CISS weakened with increasing precursor concentration. This result contradicts the notion that a thicker film should exhibit stronger selectivity. However, it can be rationalized by an increase in spin relaxation due to spin scattering during the actual film growth process. The presence of a Schottky contact between the n-type c-HP and the FM metal expands the application prospects of chiral semiconductors. Our method for quantitatively evaluating CISS using KPFM testing techniques to scan on the same location is more concise and feasible, providing new insights into understanding the CISS mechanism.

## MATERIALS AND METHODS

The detailed materials and methods are available as Supplementary data.

## Supplementary Material

nwaf295_Supplemental_File

## DATA AND MATERIALS AVAILABILITY

All data needed to evaluate the conclusions in the paper are present in the paper and/or the Supplementary data. Additional data related to this paper may be requested from the authors.
