# Supplementary material for: Spatial mapping of chiral-induced spin selectivity in chiral perovskite via spin-Schottky junction
Source: Natl Sci Rev. 2025 Jul 21;12(9):nwaf295. doi: 10.1093/nsr/nwaf295 (PMC12416287; doi:10.1093/nsr/nwaf295)
Supplement: nwaf295_Supplemental_File [file nwaf295_supplemental_file.pdf]

Supplementary Materials for

Spatial Mapping of Chiral-Induced Spin Selectivity in Chiral Perovskite via Spin-Schottky Junction

Minghui Li,[1,2#] Zhongwei Chen,[3#] Xiting Lang,[1] Junchuan Zhang,[1] Yongjie Jiang,[1] Hao Tian,[1] Fang Ye,[1,2] Xirui Liu,[1,2] Yangyang Gou,[1] Herui Xi,[1] Wei Guo,[1] Jichun Ye,[1] Matthew C. Beard,[4]* Haipeng Lu,[3]* and Chuanxiao Xiao[1,5]*

Affiliations

[1]Ningbo Institute of Materials Technology and Engineering, Chinese Academy of Sciences, Ningbo 315201, China

[2]School of Materials Science and Chemical Engineering, Ningbo University, Ningbo, Zhejiang 315211, China

[3]Department of Chemistry, The Hong Kong University of Science and Technology, Kowloon, Hong Kong (SAR) 999077, China

[4]Chemistry & Nanoscience Center, National Renewable Energy Laboratory, Golden, Colorado 80401, United States

[5]Ningbo New Materials Testing and Evaluation Center CO., Ltd, Ningbo, 315201, China

[#]M. Li and Z. Chen contributed equally to this work.

***Corresponding Authors**

E-mail: *Matt.Beard@nrel.gov*; *haipenglu@ust.hk*; *cxiao@nimte.ac.cn*.

This PDF file includes:

Materials and Methods

Figures S1 to S17

Tables S1 to S2

MATERIALS AND METHODS

Materials

All chemicals were used as received. Lead oxide (PbO) was purchased from Aladdin, (*R*)-(+)-α-methylbenzylamine (*R*-MBA), (*S*)-(−)-α-methylbenzylamine (*S*-MBA), and (±)-α-methylbenzylamine (*rac*-MBA) were purchased from Sigma-Aldrich.

The (*R*-MBA)$_2$PbI$_4$, (*S*-MBA)$_2$PbI$_4$, and (*rac*-MBA)$_2$PbI$_4$ single crystals were synthesized through a slow cooling method. 200 mg PbO, and 200 μL *R*-, *S*- or *rac*-MBA were dissolved in 6 ml HI solution in a vial under stirring at ~100 °C. The vials were then transferred to oil bath and gradually cooled down to room temperature at a rate of 2 °C/h to form needle-like crystals. The crystals were dried under vacuum overnight.

Thin Film Preparation

Quartz substrates were cleaned by sonicating in acetone and isopropyl for 15 minutes and ultraviolet-ozone treatment for 20 minutes. The precursor solution was prepared by dissolving the corresponding perovskite crystals in DMF with various concentrations. The solution was spin-coated onto the substrate at 4000 rpm for 30 s within a glovebox, followed by annealing at 80 ℃ for 10 minutes. Thin film on the quartz substrate with a precursor concentration of 0.3 mg/μL was used for absorption, X-ray diffraction, and circular dichroism spectra measurement.

Device Preparation

For the Schottky samples, 8 nm of Ti, 80 nm of Ni, and 8 nm of Au were sequentially deposited onto an *n*-type silicon wafer (1 cm × 1 cm) using electron beam deposition in a vacuum chamber at a pressure of 10^{-7} torr. The metal-deposited substrates were cleaned in boiling isopropanol for 10 minutes and ultraviolet-ozone treatment for 20 minutes. The precursor solution was prepared by dissolving the corresponding perovskite crystals in DMF with various concentrations and filtered using a PTFE filter with a pore size of 0.45 μm. A 100 μL solution was spin-coated onto the substrate at 4000 rpm for 30 s within a glovebox, followed by annealing at 80 ℃ for 10 minutes.

For the fabrication of spin valve devices, the I-V curves of the devices were tested by a Keithley 2450 source meter. Rectangular patterned bottom electrodes were deposited, and cross-patterned top Ag electrodes were fabricated by electron beam deposition under vacuum with the pressure below 3×10^{-6} torr. The devices were stored in the glove box until further measurements. The devices were magnetized by a neodymium magnet. The device area is 0.16 mm^2.

Kelvin Probe Force Microscopy (KPFM) Measurement

The KPFM used in this experiment is a home-built system based on the Bruker Dimension Icon atomic force microscope (AFM), specifically designed for measuring surface potential and assessing sample work function. KPFM operates by detecting the contact potential difference (CPD) between the probe and the sample, achieved through the continuous application of a DC voltage to nullify the coulomb force. In our setup, potential data were acquired by using external, high-performance data separation, filtering, feedback equipment, and a low-noise lock-in amplifier (Signal Recovery 7208). This approach enables simultaneous acquisition of both sample topography and surface potential information, yielding an electrical resolution exceeding 10 mV and a spatial resolution better than 30 nm. The scanning speed ranged from 0.3 to 0.45

Hz to ensure optimal image quality. A conductive probe (PPP-EFM-20) was used, featuring a Pt-Ir coating across the entire front surface to ensure excellent electrical performance.

The entire preparation and testing process were conducted within a glovebox filled with argon gas. Silver conductive adhesive was used to fix the silicon substrate with the magnet/test platform. The KPFM measurement sequence was: no additional magnetic field—north pole magnetization—south pole magnetization. The sample was magnetized for approximately 1 hour. Subsequently, KPFM testing was performed on the sample located on the magnet, and the potential information after magnetic field polarization was obtained. Notably, to eliminate the influence of sample variation across different regions, the test results were obtained from the same microscopic area in conditions of no magnetic field, North pole, and South pole environments. Additionally, to ensure measurement reliability, the probe work function was regularly validated using a known reference sample, which is to prevent the Pt-Ir coating from wearing out and leading to potential measurement artifacts. To evaluate potential probe wear, we used a silicon wafer with a known work function of 3.73 eV as a reference sample.

Magnetic Measurement

We used the 3D magnetic field tester (MMS-1A-RS) produced by SENIS to measure the magnets used in the experiment. The measure mode was the multi-pole rotation mode. The scanning resolution of the instrument is 0.1 °.

Scanning Electron Microscope（SEM）Measurement

SEM images were collected using a Hitachi S-4800 FEG SEM scanning electron microscope. The exposed cross-sections were obtained by cutting the samples with a diamond knife. Both the planar samples and the cross-sectional samples were subjected to Sputter Au treatment before testing.

X-Ray Diffraction (XRD) Measurement

XRD spectra of the thin film samples were collected on the Rigaku MiniFlex powder-X-ray diffractometer with a Cu Kα1/Kα2 source (λ = 1.54051/1.54433 Å).

UV-Vis Absorption Measurement

Absorption spectra of thin film samples were collected by UH5700 spectrophotometer. To get the Tauc plot, absorption spectra of powder samples were collected by diffuse reflectance mode. $BaSO_4$ was used as the 100% reflectance background. The Kubelka−Munk equation, $\alpha/S = (1 - R)^2/(2R)$, where R is the reflectance, α is the absorption coefficient, and S is the scattering coefficient, was applied to convert the reflectance to the absorption data.

Circular Dichroism (CD) Measurement

CD spectra of the thin films were all collected by Applied Photophysics Chirascan at room temperature.

Ultraviolet Photoelectron Spectroscopy (UPS) Measurement

UPS spectra were measured by an Axis Supra+ surface analysis system with a UV excitation of 21.22 eV.

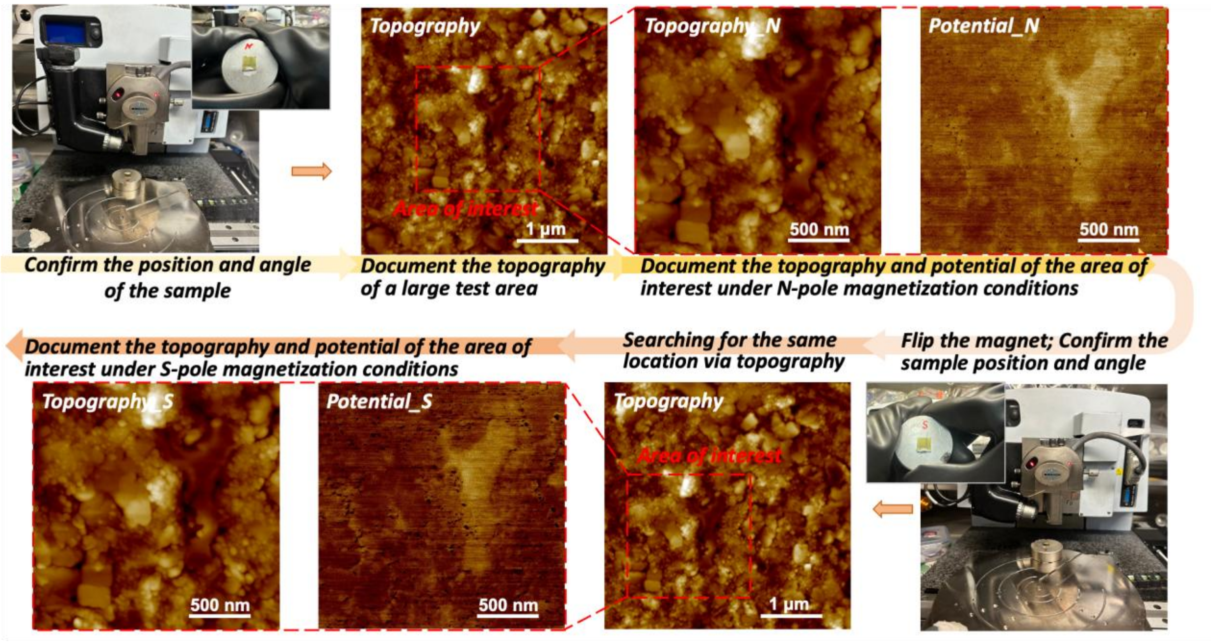

Figure S1. The operation procedure for KPFM testing on the same location.

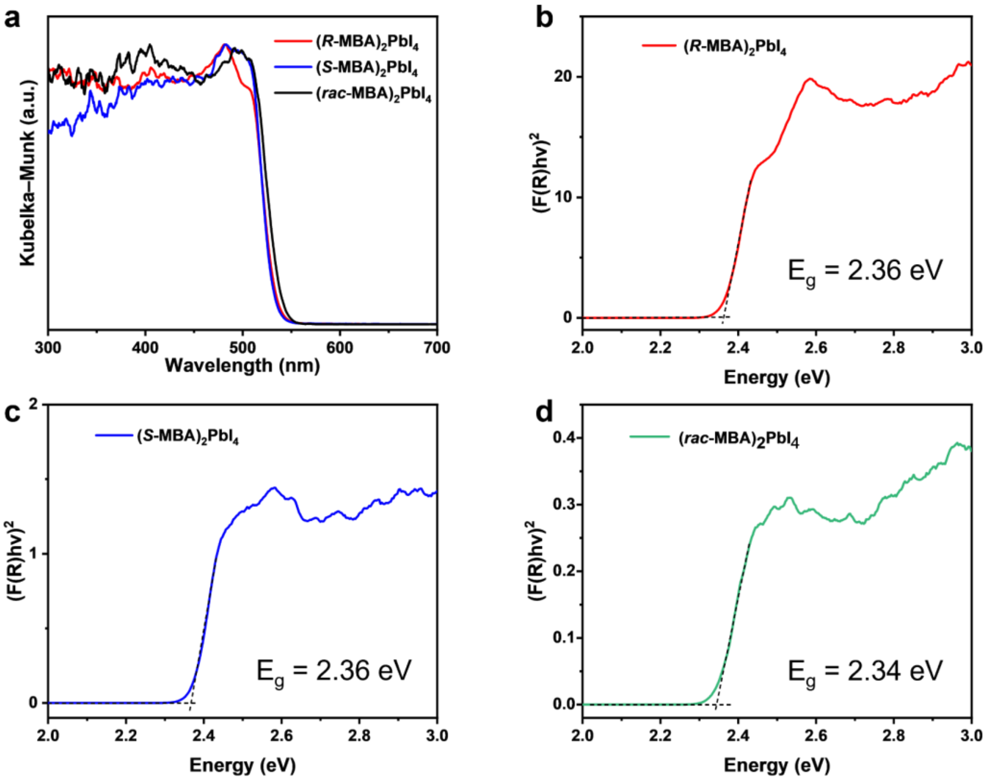

Figure S2. (a) Absorption spectra of (*R*-/*S*-/*rac*-MBA)$_2$PbI$_4$. (b-d) Tauc plots of (*R*-MBA)$_2$PbI$_4$, (*S*-MBA)$_2$PbI$_4$, and (*rac*-MBA)$_2$PbI$_4$, respectively.

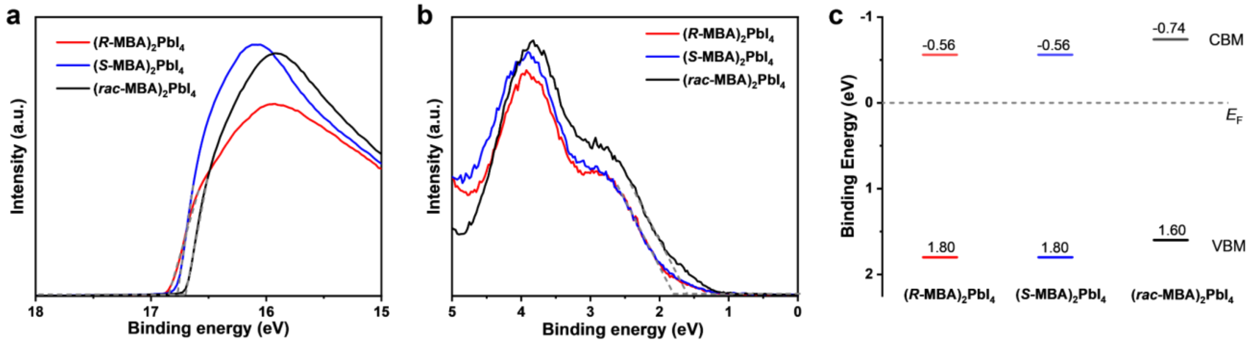

Figure S3. (a) UPS of the cut-off region. (b) UPS spectra of the valence band edge region. (c) Energy levels of the (*R*-/*S*-/*rac*-MBA)$_2$PbI$_4$.

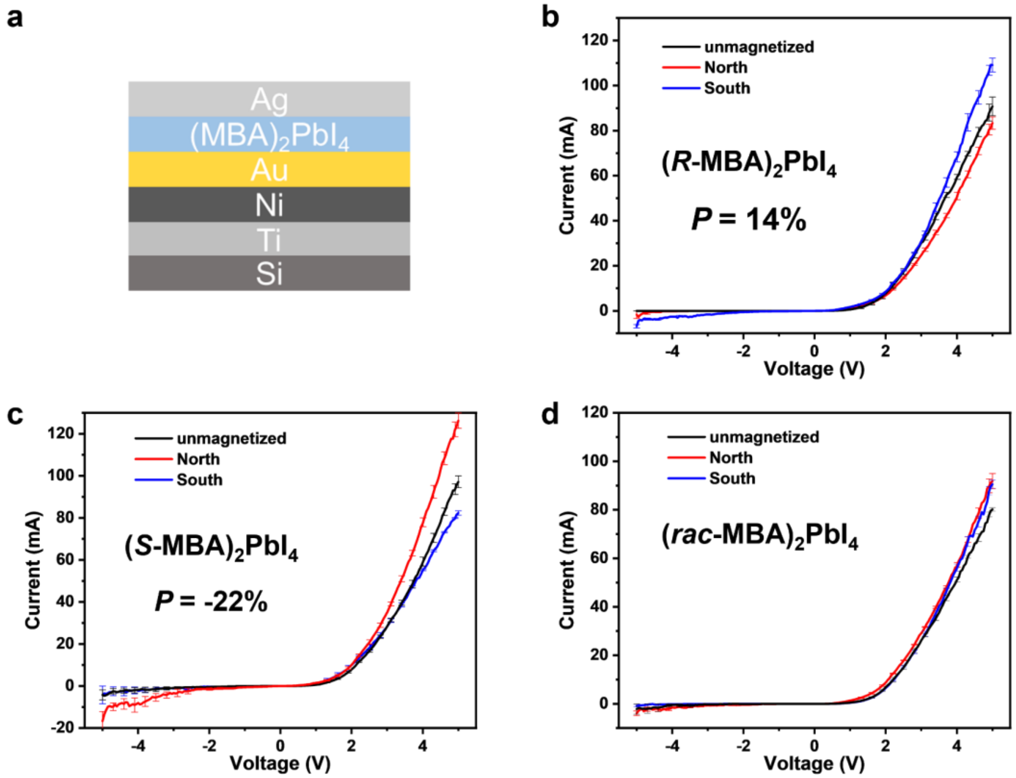

Figure S4. (a) The structure of fabricated CISS devices. (b-d) I-V curves of (*R*-MBA)$_2$PbI$_4$, (*S*-MBA)$_2$PbI$_4$ and (*rac*-MBA)$_2$PbI$_4$, respectively. We define the spin polarization (P) as: $P = (I_+ - I_-)/(I_+ + I_-) \times 100\%$, where I_+ and I_- represent the measured currents at 5 V bias under tip magnetic field orientations "South" and "North", respectively.

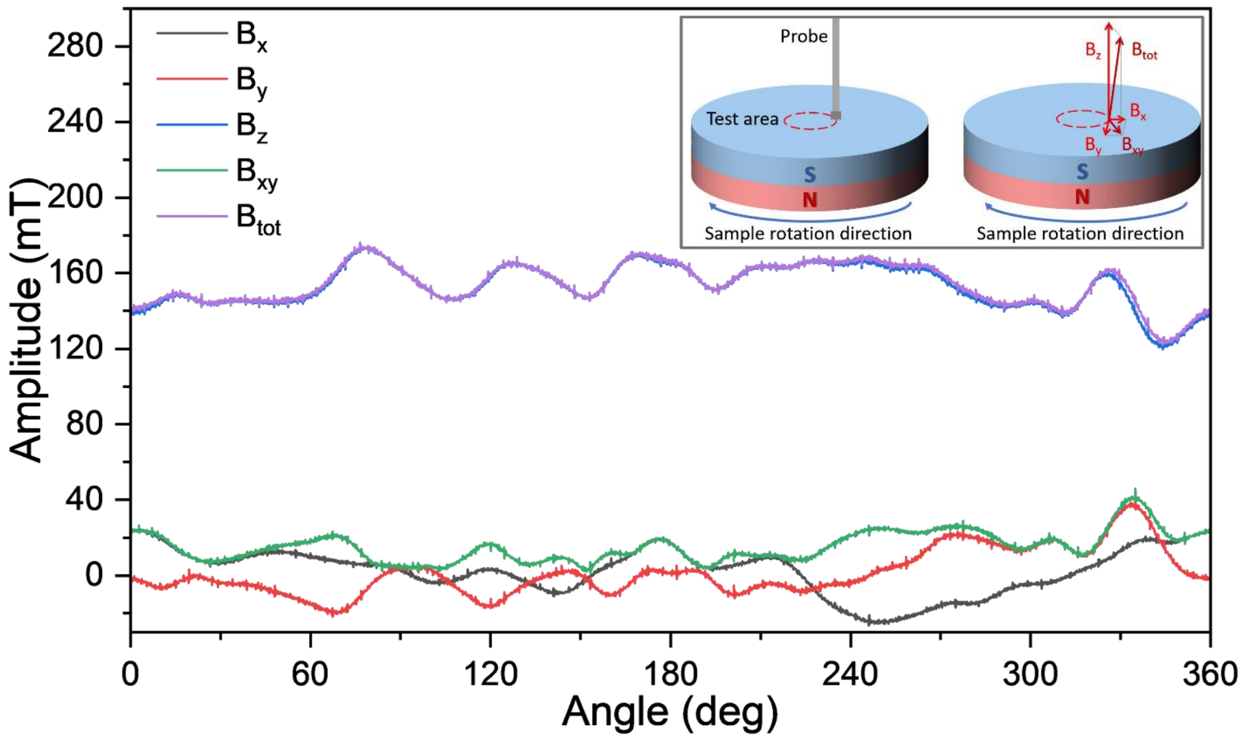

Figure S5. Magnetic field strength. The magnetization test results of the magnet used for magnetizing the chiral sample. B_x, B_y, and B_z represent the magnetic field components along the X-axis, Y-axis, and Z-axis, respectively. B_{xy} is the sum of the magnetic field components in the X and Y directions, and B_{tot} is the total magnetic field strength. The inset illustration presents a schematic diagram of the 3D magnetic field tester model, and the different magnetic field components (B_x, B_y, B_z, B_{xy}, B_{tot}).

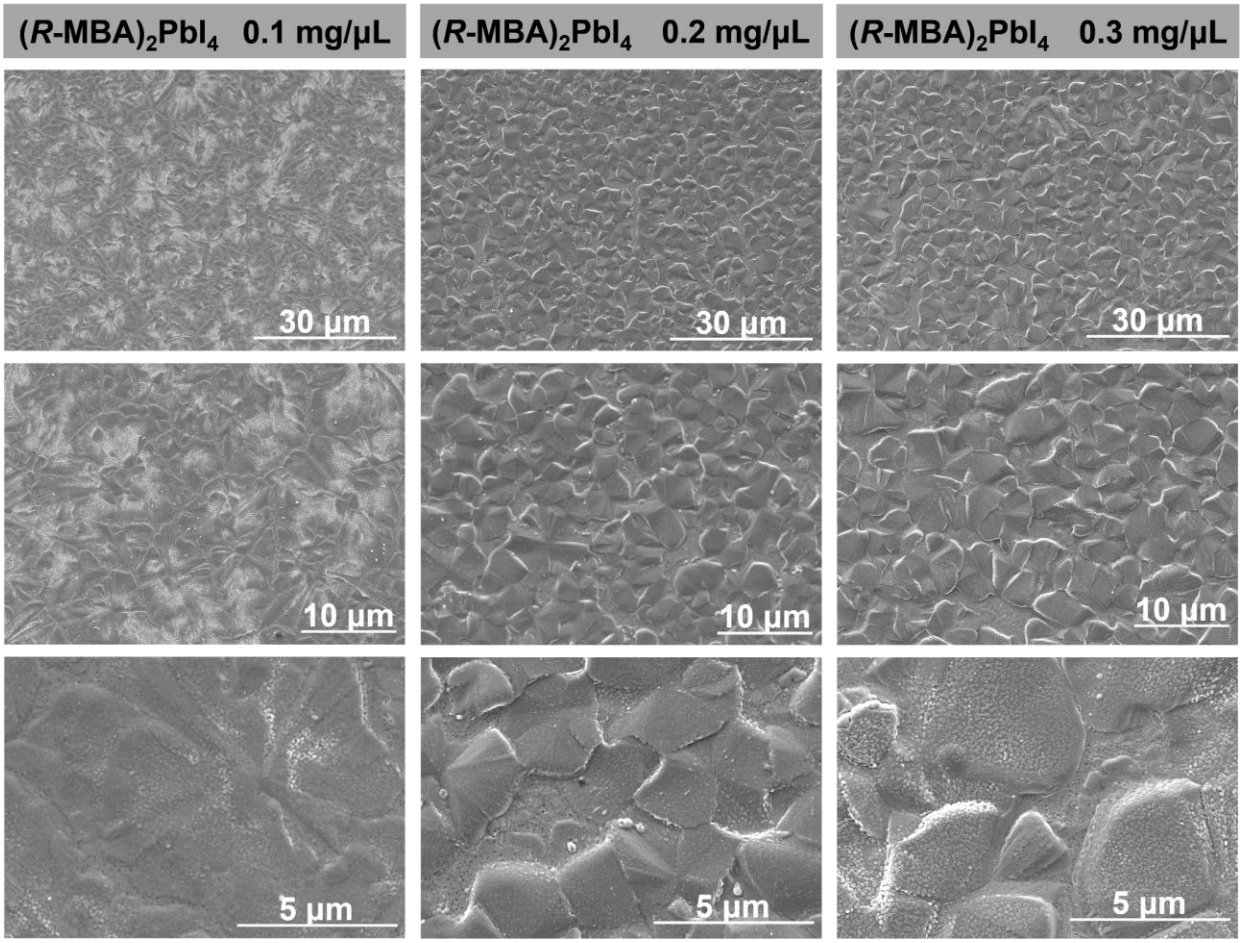

Figure S6. SEM images of (R-MBA)$_2$PbI$_4$ thin films at different concentrations under varying magnifications.

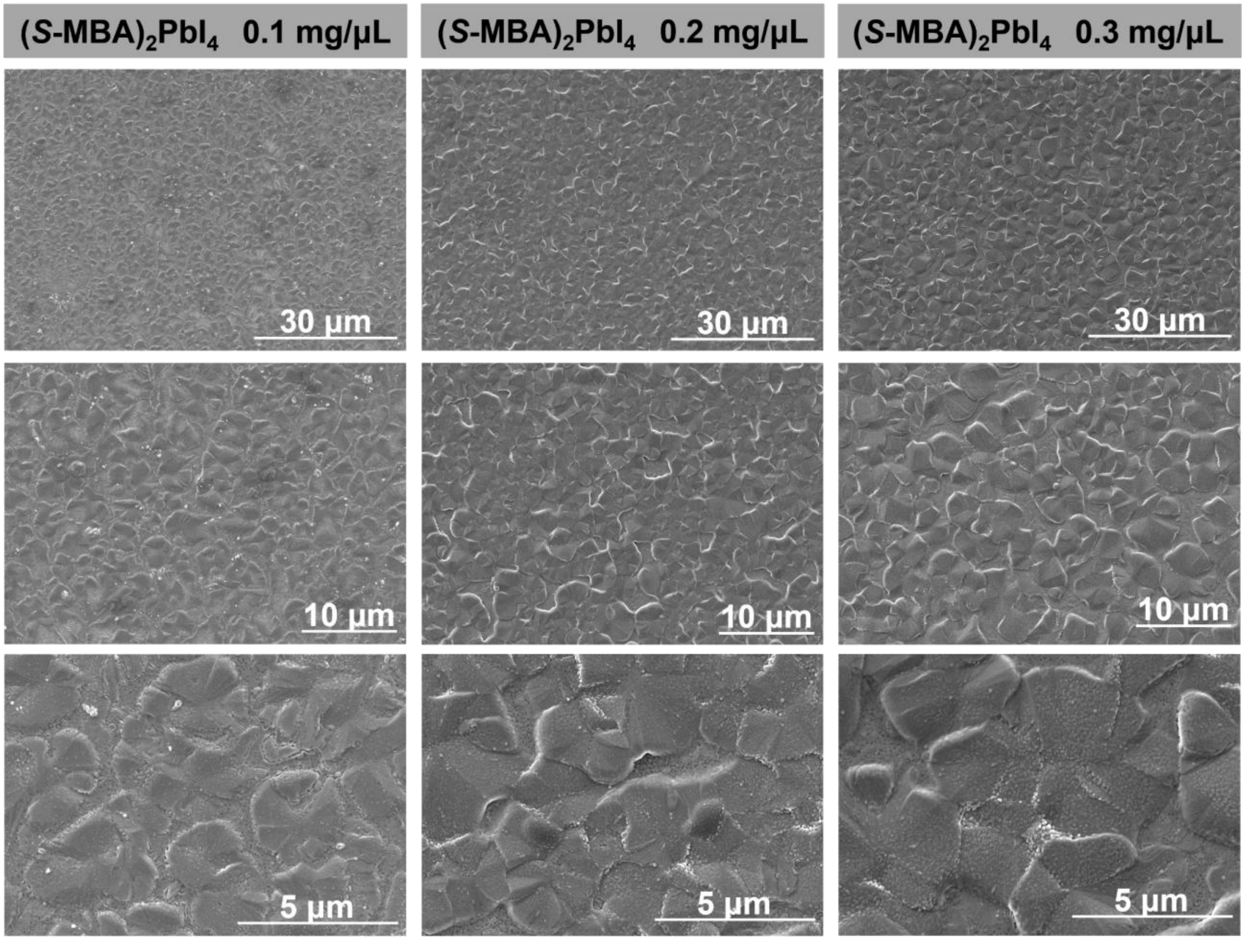

Figure S7. SEM images of (S-MBA)$_2$PbI$_4$ thin films at different concentrations under varying magnifications.

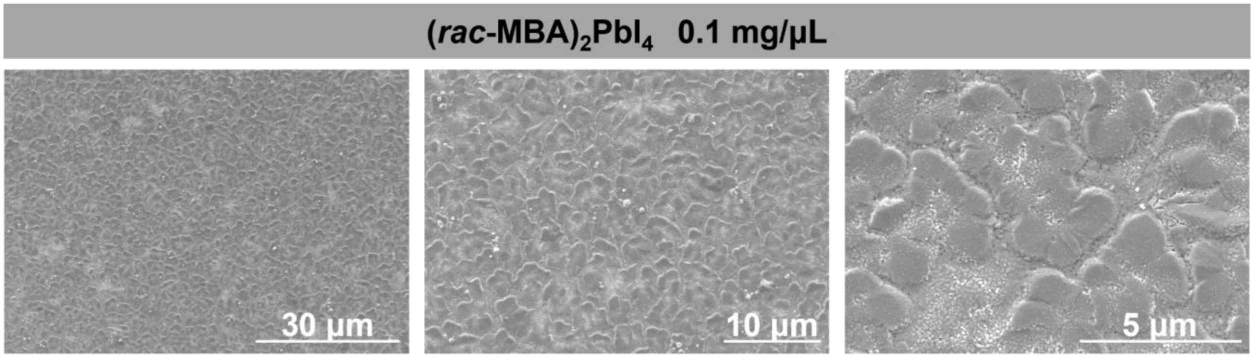

Figure S8. SEM results of (*rac*-MBA)$_2$PbI$_4$ thin films at a concentration of 0.1 mg/μL under different magnifications.

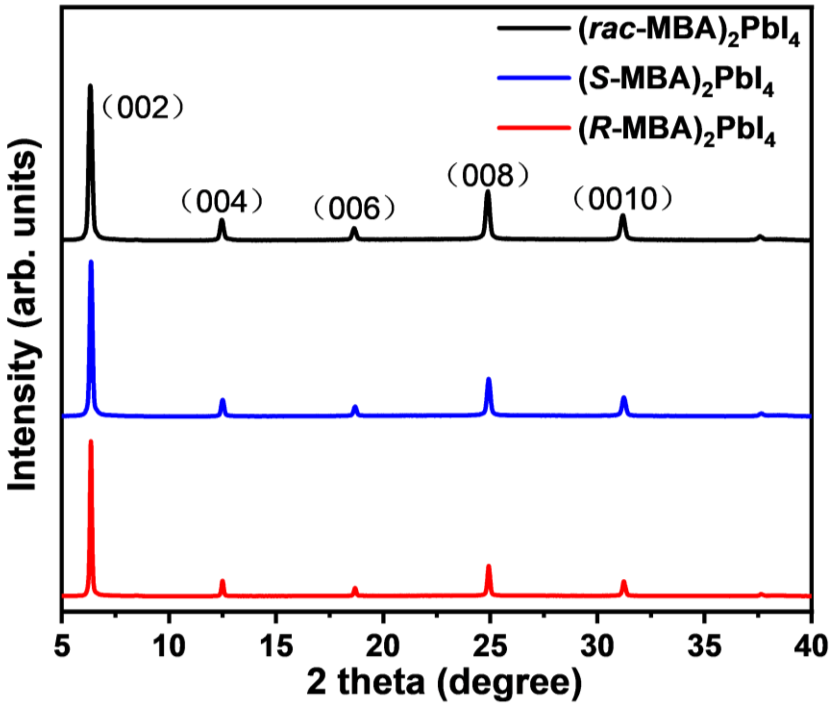

Figure S9. XRD patterns of the 2D perovskite thin films.

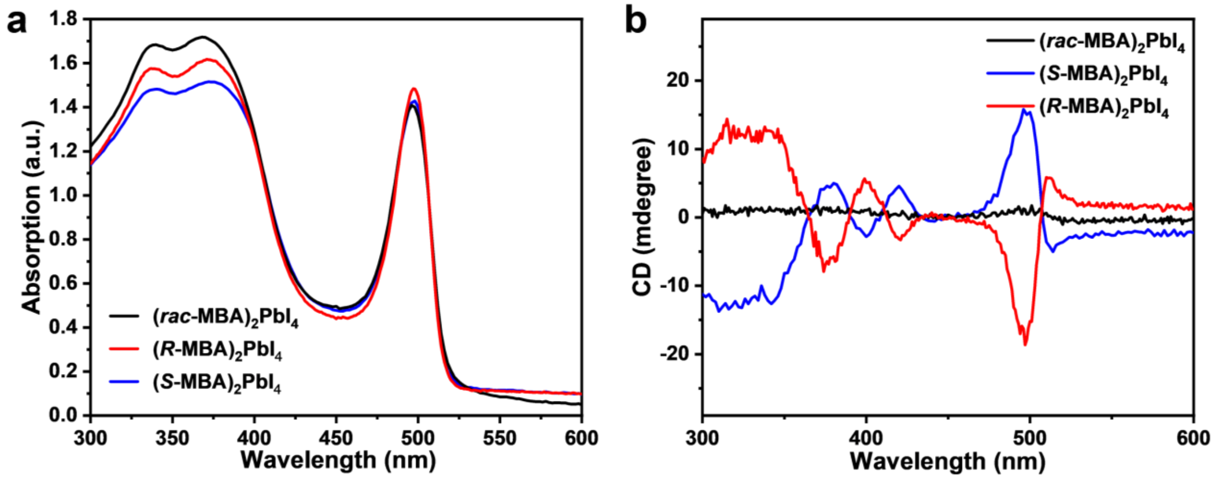

Figure S10. (a) (b) Absorption and CD spectra of the 2D perovskite thin films.

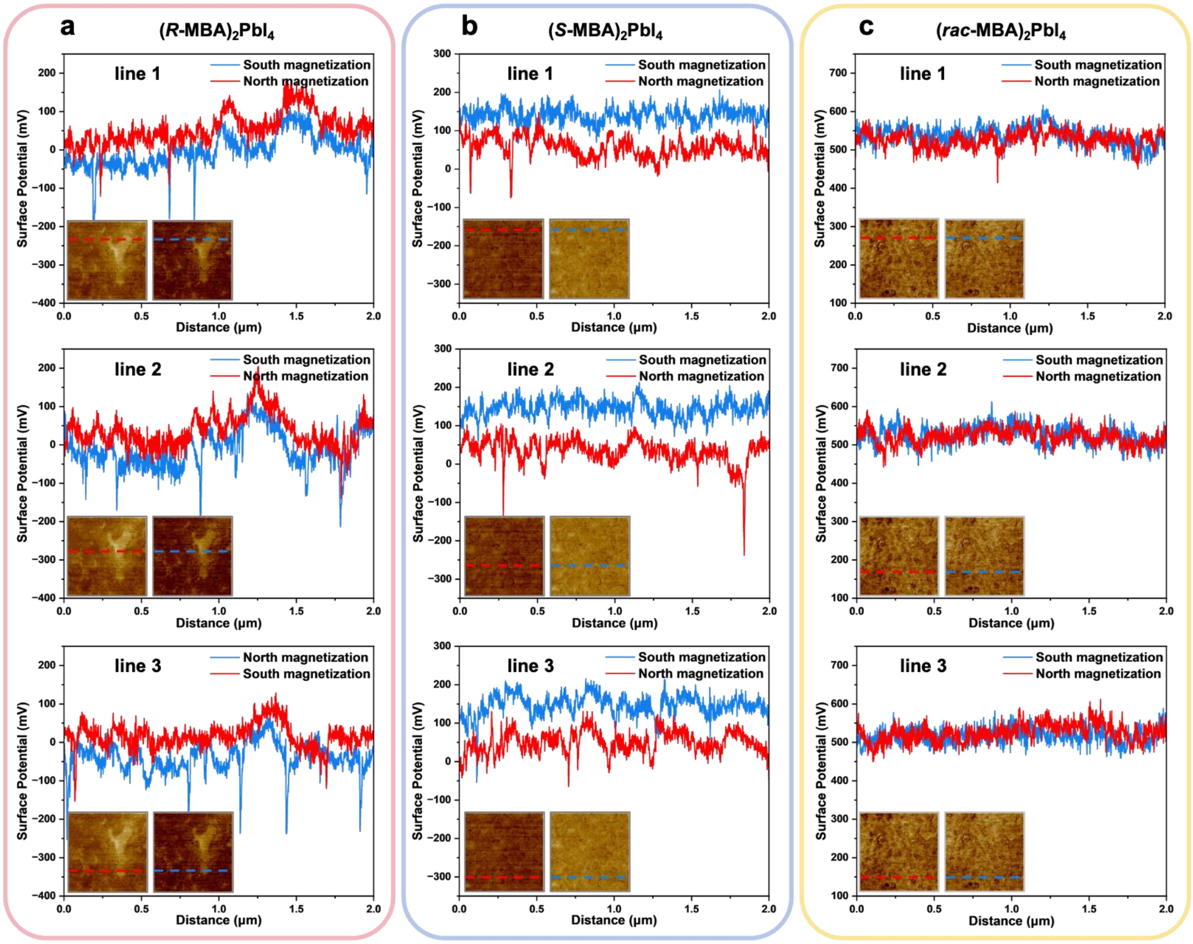

Figure S11. KPFM line profiles under different magnetic field conditions. (a-c) line profiles of (*R*-/*S*-/*rac*-MBA)$_2$PbI$_4$. The dash lines in the inset indicate where the line profiles were taken.

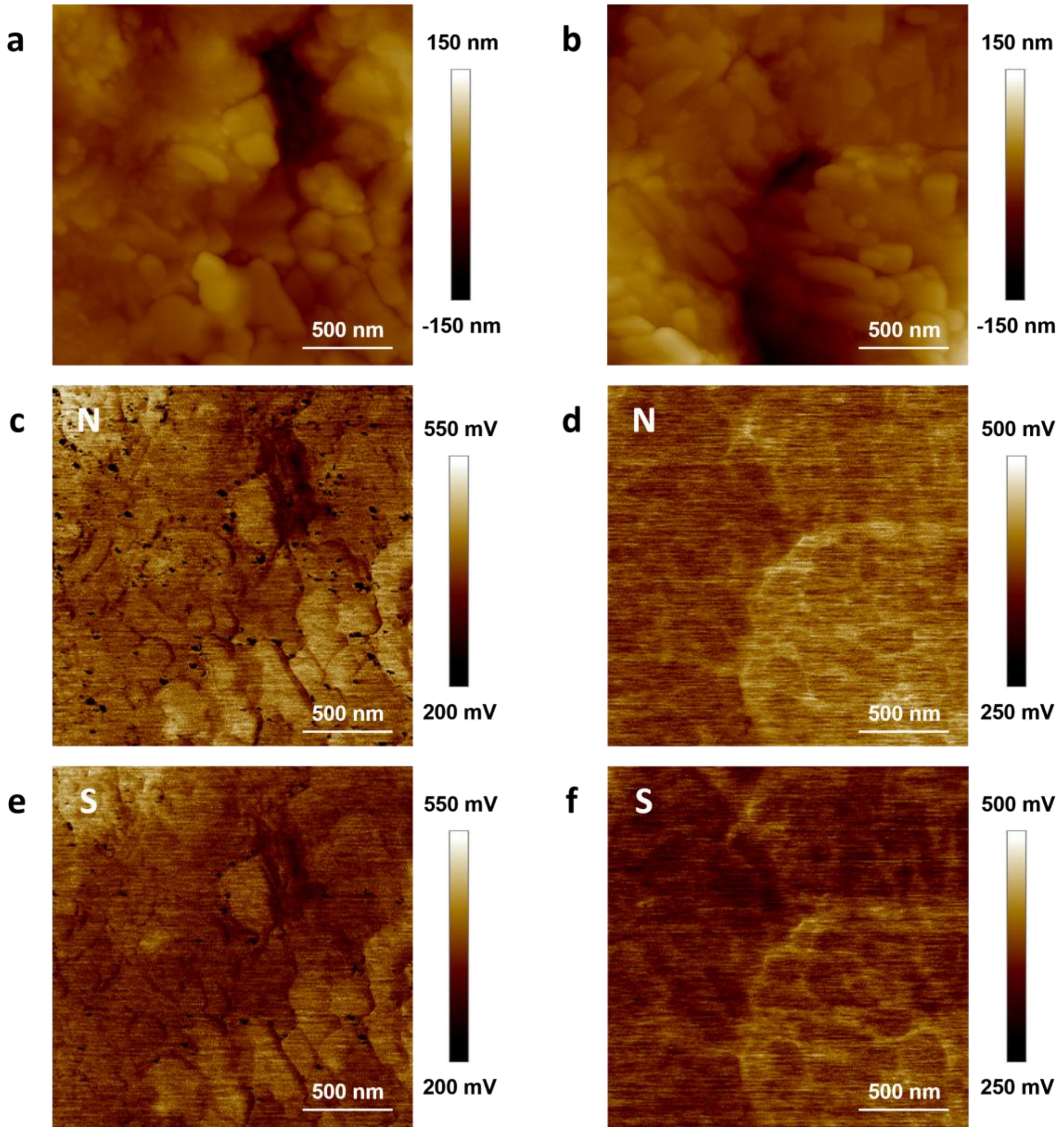

Figure S12. The KPFM test results of (R-MBA)$_2$PbI$_4$ materials at different concentrations. (a, b) AFM results of concentrations 0.2 mg/μL and 0.3 mg/μL. (c, d) KPFM results under North magnetization condition for concentrations 0.2 mg/μL and 0.3 mg/μL. (e, f) KPFM results under South magnetization condition for concentrations 0.2 mg/μL and 0.3 mg/μL.

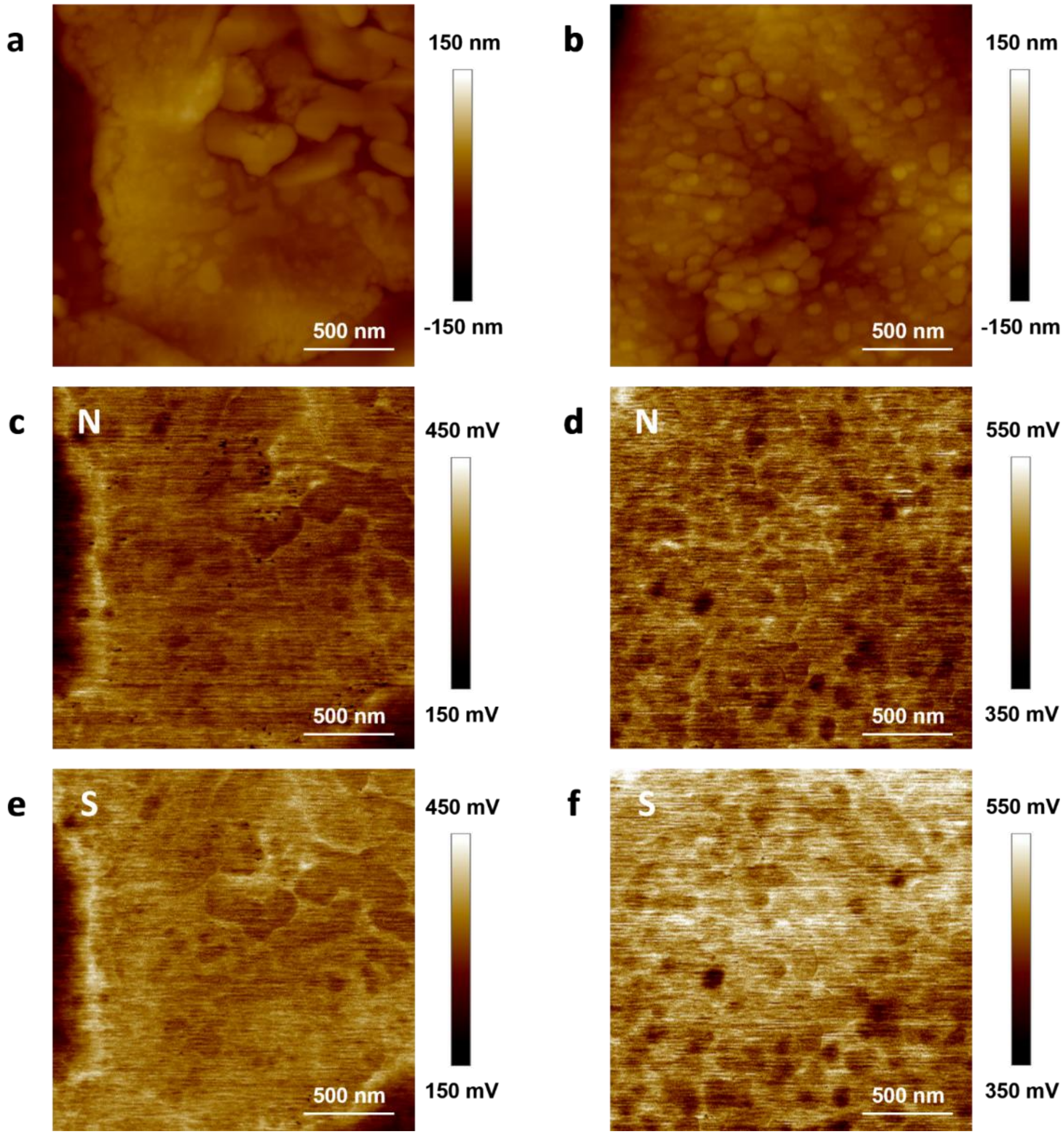

Figure S13. The KPFM test results of (S-MBA)$_2$PbI$_4$ materials at different concentrations. (a, b) AFM results of concentrations 0.2 mg/μL and 0.3 mg/μL. (c, d) KPFM results under North magnetization condition for concentrations 0.2 mg/μL and 0.3 mg/μL. (e, f) KPFM results under South magnetization condition for concentrations 0.2 mg/μL and 0.3 mg/μL.

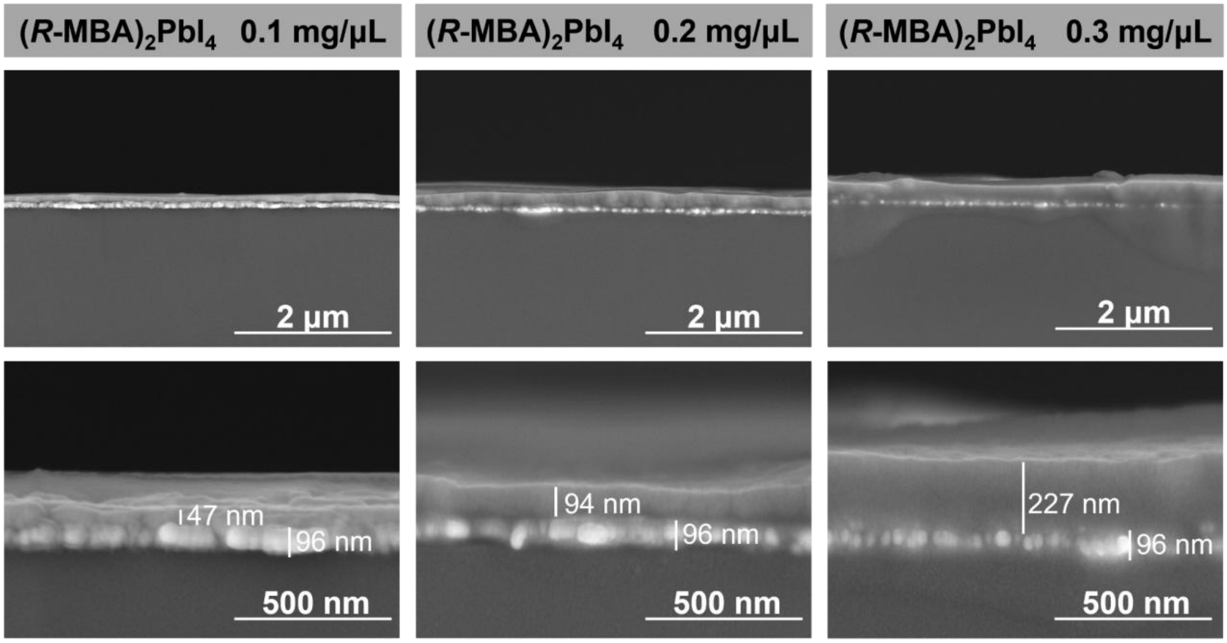

Figure S14. Cross-sectional SEM results of (R-MBA)$_2$PbI$_4$ samples at different concentrations under varying magnifications.

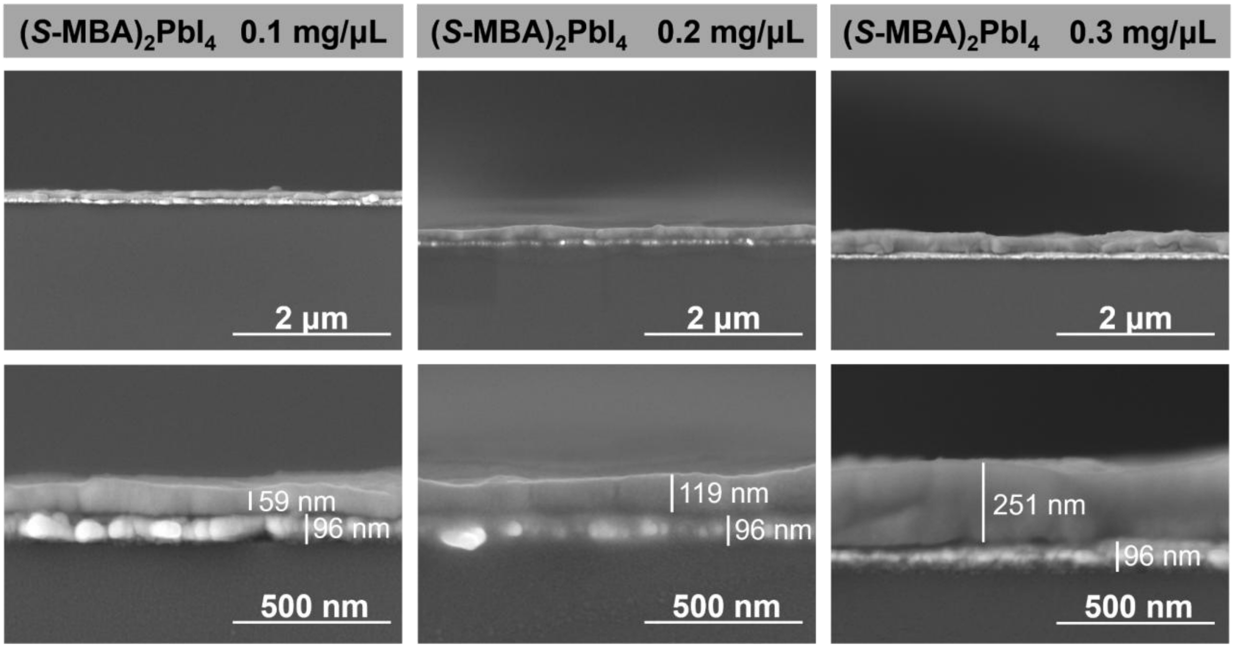

Figure S15. Cross-sectional SEM results of (*S*-MBA)$_2$PbI$_4$ samples at different concentrations under varying magnifications.

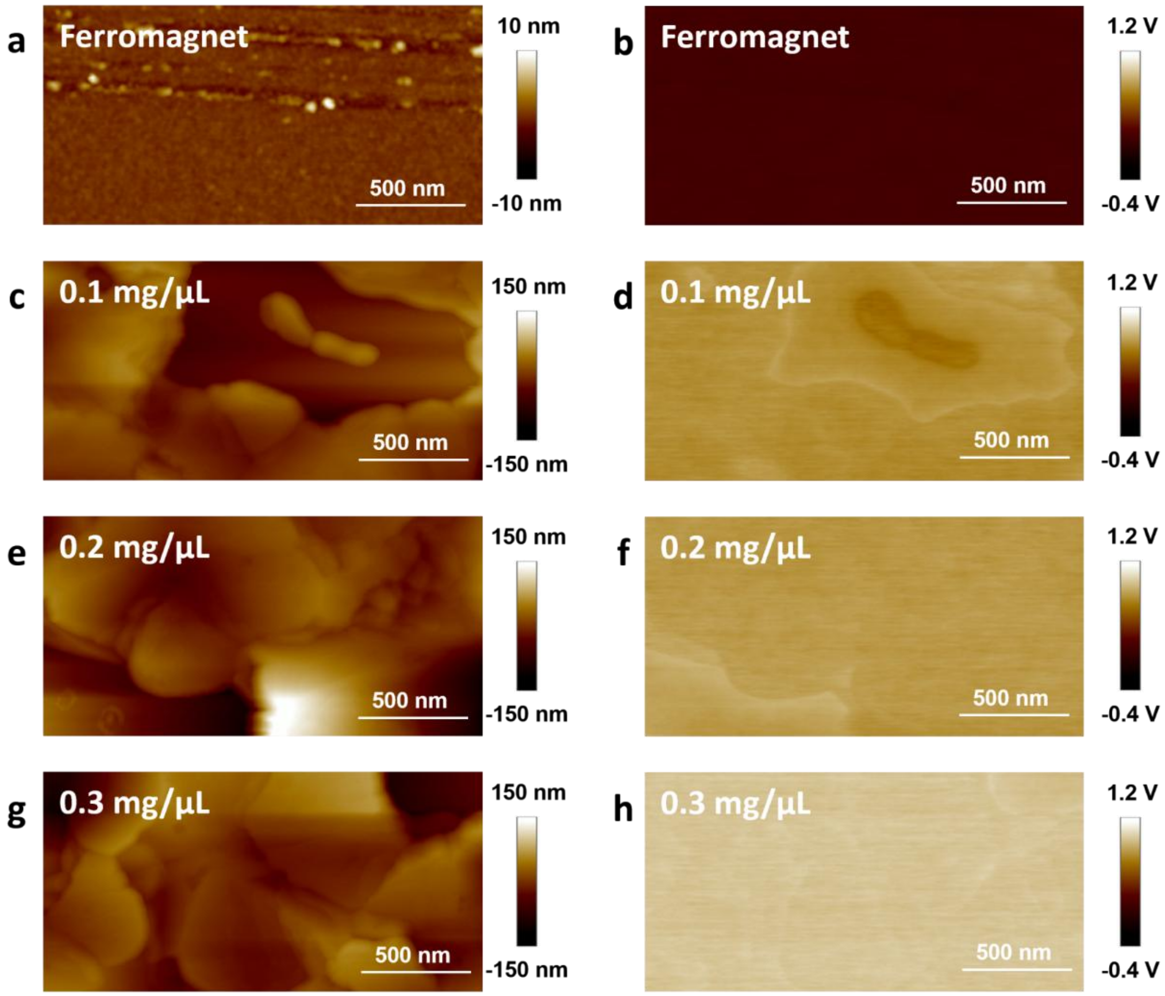

Figure S16. KPFM results of (*R*-MBA)$_2$PbI$_4$ materials and pure ferromagnetic substrate. (a) AFM results of pure ferromagnetic substrate. (b) KPFM results of pure ferromagnetic substrate. (b, e, g) AFM results of concentrations 0.1 mg/μL, 0.2 mg/μL, and 0.3 mg/μl. (d, f, h) KPFM results of concentrations 0.1 mg/μL, 0.2 mg/μL, and 0.3 mg/μL.

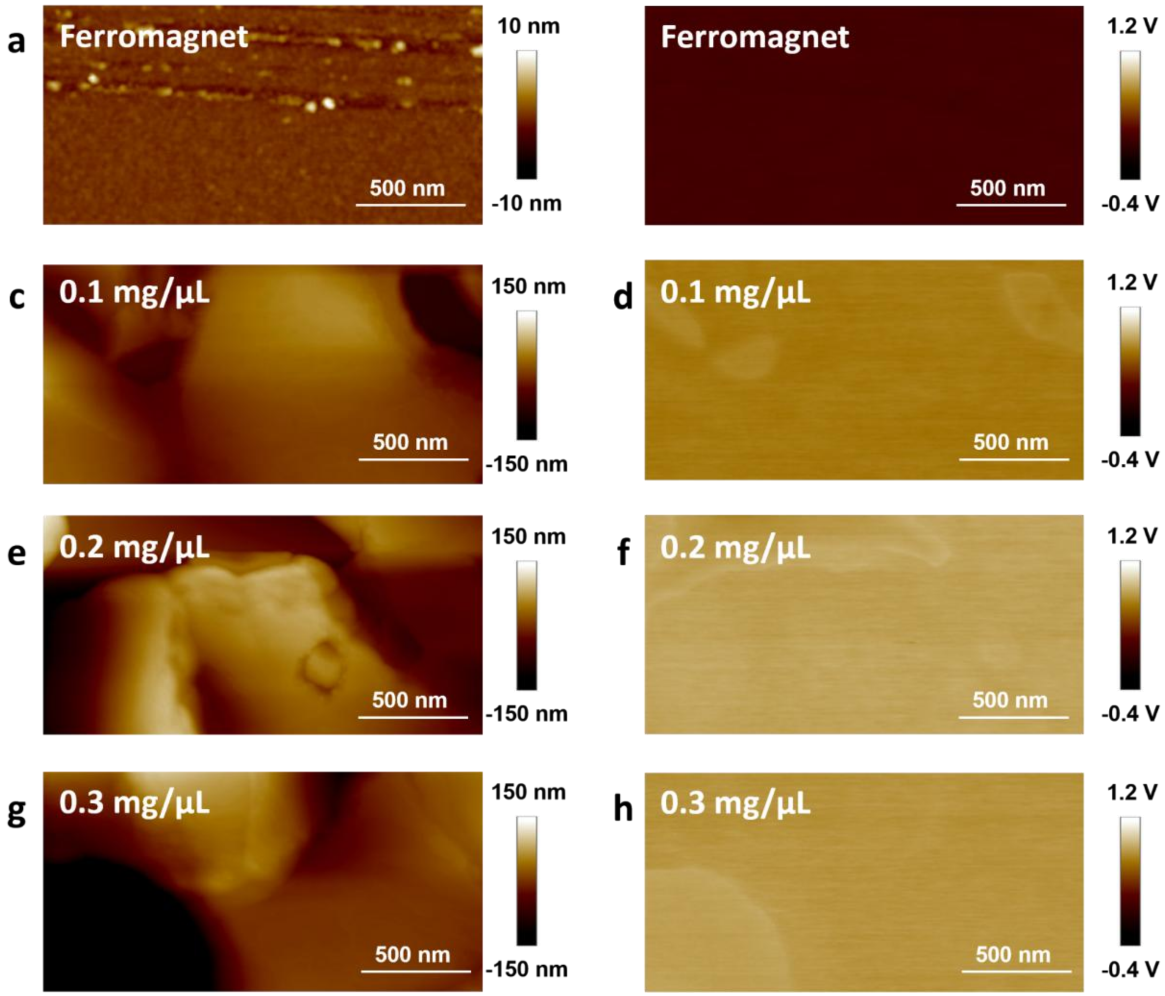

Figure S17. KPFM results of (*S*-MBA)$_2$PbI$_4$ materials and pure ferromagnetic substrate. (a) AFM results of pure ferromagnetic substrate. (b) KPFM results of pure ferromagnetic substrate. (b, e, g) AFM results of concentrations 0.1 mg/μL, 0.2 mg/μL, and 0.3 mg/μL. (d, f, h) KPFM results of concentrations 0.1 mg/μL, 0.2 mg/μL, and 0.3 mg/μL.

Table S1. The half-width at half maximum of the normalized potential peaks calculation results of (R-MBA)$_2$PbI$_4$ materials at different concentrations under various magnetization conditions.

(R-MBA)$_2$PbI$_4$	(0.1 mg/μL)	(0.2 mg/μL)	(0.3 mg/μL)
North	40.3 mV	26.3 mV	27.4 mV
South	44.5 mV	37.5 mV	28.9 mV

Table S2. The half-width at half maximum of the normalized potential peaks calculation results of (S-MBA)$_2$PbI$_4$ materials at different concentrations under various magnetization conditions.

(S-MBA)$_2$PbI$_4$	(0.1 mg/μL)	(0.2 mg/μL)	(0.3 mg/μL)
North	43.6 mV	27.0 mV	23.1 mV
South	30.5 mV	23.6 mV	29.7 mV
